# Improved Ferroelectric and Magnetic Properties of Bismuth Ferrite-Based Ceramics by Introduction of Non-Isovalent Ions and Grain Engineering

**DOI:** 10.3390/nano15030215

**Published:** 2025-01-29

**Authors:** Ting Wang, Huojuan Ye, Xiaoling Wang, Yuhan Cui, Haijuan Mei, Shenhua Song, Zhenting Zhao, Meng Wang, Pitcheri Rosaiah, Qing Ma

**Affiliations:** 1 Guangdong Provincial Key Laboratory of Electronic Functional Materials and Devices, Huizhou University, Huizhou 516001, China; 2 School of Mechanical and Electrical Engineering, Hebei Vocational College of Rail Transportation, Shijiazhuang 050801, China; cuiyhan@126.com; 3 School of Materials Science and Engineering, Harbin Institute of Technology (Shenzhen), Shenzhen 518055, China; 4 School of Undergraduate Education, Shenzhen Polytechnic University, Shenzhen 518055, China; 5 Department of Physics, Saveetha School of Engineering, Saveetha Institute of Medical and Technical Sciences (SIMATS), Thandalam, Chennai 602105, India; rosaiah.p@gamil.com

**Keywords:** bismuth ferrite, spark plasma sintering, Nd–Nb co-doping, ferroelectric property, magnetic property

## Abstract

Single-phase multiferroics exhibiting ferroelectricity and ferromagnetism are considered pivotal for advancing next-generation multistate memories, spintronic devices, sensors, and logic devices. In this study, the magnetic and electric characteristics of bismuth ferrite (BiFeO_3_) ceramics were enhanced through compositional design and grain engineering. BiFeO_3_ ceramic was co-substituted by neodymium (Nd) and niobium (Nb), two non-isovalent elements, via the spark plasma sintering process using phase-pure powder prepared via sol-gel as the precursor. The symmetry of the sintered Nd–Nb co-doped samples changed from *R*3*c* to *Pnma*, accompanied by a decrease in the loss tangent, grain size, and leakage current density. The reduction in the leakage current density of the co-doped samples was ~three orders of magnitude. Moreover, ferroelectric, dielectric, and magnetic properties were substantially improved. The remanent polarization and magnetization values of the optimized Nd–Nb co-doped BiFeO_3_ sample were 3.12 μC cm^−2^ and 0.15 emu g^−1^, respectively. The multiferroic properties were enhanced based on multiple factors such as structural distortion caused by co-doping, grain size reduction, suppression of defect charges via donor doping, space-modulated spin structure disruption, and an increase in magnetic ions. The synergistic approach of composition design and grain engineering sets a paradigm for the advancement of multiferroic materials.

## 1. Introduction

Compounds with ≥2 primary ferroic orders (such as ferromagnetism, ferroelasticity, and ferroelectricity) exhibit multiferroic characteristics. These compounds have been technologically and economically important over the past two decades, and continue to be now and will be in the future [1]. The effective magnetoelectric coupling at room temperature has several applications in data storage systems, adjustable microwave components, sensors, and random-access memory devices [2,3,4]. Magnetoelectric multiferroic materials are of three types: (i) single-phase type I multiferroics with distinct magnetization and polarization origin, (ii) single-phase type II multiferroics with polarization induced by a particular magnetic configuration, and (iii) composite materials or heterostructures (piezoelectric multilayer and artificial ferromagnetic capacitors) with strain-mediated interactions modulating magnetic properties under an electric field (*E*) [1].

Bismuth ferrite (BiFeO_3_, BF) has emerged as the quintessential example of single-phase type I multiferroic materials at room temperature since 2003 owing to its substantial ferroelectric polarization and elevated phase transition temperatures (Curie temperature (*T*_C_) of ~825 °C and Néel temperature (*T*_N_) of ~370 °C) [5]. It exhibits a distorted rhombohedral perovskite structure belonging to the *R*3*c* space group with lattice parameter values of length (*a*) and angle (*α*) of 3.958 Å and 89.30°, respectively [6]. It is characterized by better ferroelectric properties than lead-free materials such as bismuth titanate (Bi_4_Ti_3_O_12_) [7], with simultaneous antiferromagnetic and ferroelectric characteristics at ambient temperature [6]. BF crystals exhibit ferromagnetism and ferroelectricity owing to the partially filled 3D orbits of ferric ions (Fe^3+^) and the stereochemically active lone-pair electrons in the 6s^2^ orbitals of bismuth ions (Bi^3+^) [8]. The theoretical ferroelectric polarization value of BF in the [111] direction is in the range of 90–100 μC cm^−2^ [9], while the magnetoelectric effect is prominent with the coupling energy of ~1.25 meV [10]. Although exceptional electrical properties of BF are predicted theoretically, it is practically challenging owing to the high leakage current density (*J*). This is attributed to oxygen vacancy, Bi evaporation, the formation of the frequent secondary phase, the presence of mixed Fe^3+^ and ferrous (Fe^2+^) ions, and weak ferromagnetism, which substantially limit its application in various sophisticated devices [10]. The central challenge for BiFeO_3_-based multiferroic ceramics has been to concurrently enhance ferroelectricity and ferromagnetism, thereby improving the magnetoelectric coupling.

Chemical substitution at the A-, B-, or A/B-sites of BF is an effective method to increase its ferroelectric properties by reducing leakage current [1,2,3,4,11]. Lakshmi et al. [12] reported a substantial reduction in the leakage current of BF ceramics doped with niobium (Nb) of high valency. The doping of Nb could alter the Fe-O-Fe bond angle, potentially disrupting the spin cycloid structure. Abe et al. [13] examined the effects of cation substitution with different valences in BiFeO_3_ ceramics and observed a marked reduction in leakage current by substituting BF with 10% titanium ion (Ti^4+^), which was attributed to a decrease in oxygen vacancies. Yamamoto et al. [14] identified a transition from a cycloidal spin structure to a collinear spin structure of BF polycrystalline ceramics substituted by cobalt (Co), which induced spontaneous magnetization at room temperature owing to the spin-canting phenomenon occurring in collinear structures. The doping of A-sites with rare-earth ions is a beneficial strategy. Studies indicated that the crystal structure was stabilized via the partial replacement of the Bi^3+^ ions of BF with rare-earth ions by enhancing ionic bond strength, which suppressed secondary phases and oxygen vacancies primarily formed by the evaporation of Bi. Moreover, magnetocrystalline anisotropy increased, which was responsible for the energetically unfavorable spin cycloid structure [15,16,17,18,19,20]. Recently, the symmetry modulation of *R*3*c* to *Pna*2_1_ and eventually to *Pbnm* with the increasing substitution of lanthanum (La)- [19], neodymium (Nd)- [16], samarium (Sm)- [20], gadolinium (Gd)- [18], and La/lutetium (Lu) [17] was developed as an effective method to improve the multiferroic characteristics of BF ceramics. The emergence of the *Pna*2_1_ polar phase under chemical pressure is a determining factor in this process. Quan et al. [15] reported the improved magnetic properties of Bi_0.8_Ce_0.2_FeO_3_, which were attributed to the high canting angle and repressed cycloidal spin structure of the cerium (Ce)-doped sample. Nonzero magnetization was observed in La-substituted BF [21], a phenomenon owing to the chemical compression-induced rotation of iron oxide (FeO_6_) octahedra. In the FeO_6_ octahedra, each Fe^3+^ is surrounded by six O^2−^ and the two nearest neighboring Fe^3+^ are connected by an O^2−^, where the Fe-3d energy level and O-2p energy level hybridizing contributes to double-exchange interaction. The Fe-O-Fe bond angle changed with the doping La element, which was helpful for the exchange interaction by enhancing the overlap of the Fe 3d-O 2p orbitals. Thus, these modified the symmetry structure and suppressed the spin cycloid. Thus, the presence of rare-earth ions alters chemical pressure and disrupts the spin cycloid, progressively enhancing magnetic properties.

Hence, it was anticipated that co-doping the Fe and Bi sites of BF might decrease *J* while simultaneously enhancing magnetic and ferroelectric properties. Ke et al. [22] co-doped BF ceramics using Nd and titanium (Ti) to obtain structures with morphotropic phase boundaries (MPB) using a solid-state sintering method. The synthesized samples exhibited improved magnetic and ferroelectric properties at the boundaries with the highest remanent magnetization (*M*_r_) and polarization (*P*_r_) values of 0.27 emu g^−1^ and 29 μC cm^−2^, respectively. Gumiel et al. [23] reported that antiferromagnetism could be converted to weak ferromagnetism via co-doping (Nd-Ti), which also decreased electric conductivity, increased the electric polarization, and stabilized the converted state. Wang et al. [24] co-doped BF ceramics with Sm and Nb via spark plasma sintering and the sol-gel process. The doped samples exhibited reduced *J* with enhanced magnetic and ferroelectric properties. Gao et al. [25] reported the synthesis of polar nanoregions by co-doping BF with Sm and scandium (Sc), which increased the dielectric breakdown field. Nonetheless, reports on the improved magnetic and ferroelectric properties and the leakage current in BiFeO_3_ ceramics co-doped with Nd and high-valence Nb are scarce.

On the other hand, the technique used to fabricate ceramics significantly influences their macroscopic multiferroic characteristics. Several methods, such as rapid liquid-phase sintering [26], the sol-gel method [27], electric current activated sintering [28], flash sintering [29], and microwave sintering [30] were used to prepare single-phase BF. The spark plasma sintering (SPS) method is considered a more efficient technique than the aforementioned techniques for synthesizing materials with micro-/nanostructures that can be modulated. The pulsed electric current and uniaxial stress generated during SPS facilitate rapid sintering and effective heat transfer, inducing enhanced ceramic compaction within a short duration [31]. It is characterized by low sintering temperatures and less holding time, which inhibit Bi volatilization and the valence state change of Fe. SPS was used to successfully fabricate highly dense BF and BF-based ceramics with a pure phase and enhanced multiferroic and dielectric properties [24,31,32]. In this context, the synergistic impact of co-substitution in ferroelectric ceramics can be beneficial for enhancing physical properties. This inspired us to use SPS to prepare nanocrystalline BiFeO_3_ ceramics co-doped with rare-earth Nd^3+^ and high-valence Nb^5+^.

In this study, inspired by the concept of the grain size and ionic radius effect, the proposed strategy involves the co-substitution of non-isovalent ions (Nd and Nb) at the A/B sites via the sol-gel method followed by SPS to enhance the ferroelectric and magnetic characteristics of BF ceramics. The Bi_1−*x*_Nd*_x_*Fe_0.95_Nb_0.05_O_3_ (*x* = 0, 0.05, 0.10, 0.15, and 0.20) ceramic samples were sintered using phase-pure sol-gel-derived precursor powders. The impact of Nd–Nb co-doping on the ferroelectric, structural, dielectric, and magnetic characteristics of the synthesized samples was thoroughly investigated.

## 2. Experimental Procedure

BF and Bi_1−*x*_Nd*_x_*Fe_0.95_Nb_0.05_O_3_ (*x* = 0.05, 0.10, 0.15, and 0.20) nanopowder samples in a pure phase were synthesized using the sol-gel method, as documented in our earlier studies [33,34]. These as-synthesized powder samples were loaded into a 10 mm-diameter graphite mode and sintered via SPS. SPS was performed following the reported procedure [24,35]. The pellet was sintered at 700 °C, with a heating and cooling rate of 100 °C/min. The temperature was held there for 5 min under a vacuum of 10^−2^ Pa. A uniaxial and stable stress of 50 MPa was maintained both in the heating and soaking processes. The Bi_1−*x*_Nd*_x_*Fe_0.95_Nb_0.05_O_3_ samples with *x* values of 0.05, 0.10, 0.15, and 0.20 were denoted as 5NdNb, 10NdNb, 15NdNb, and 20NdNb, respectively.

The phase composition of the sintered ceramic samples via SPS was examined via X-ray diffraction (XRD). The microstructures of the fractured surfaces of the ceramic samples were analyzed using a field emission–scanning electron microscope (FE-SEM, model Hitachi S-4700). The samples were ground to obtain 1 mm thickness, coated with silver electrodes, and subsequently annealed for 2 h at 120 °C to analyze the dielectric properties using a computerized impedance analyzer (PSM1735, Newton 4th Ltd., UK). Polarization versus electric field (*P*–*E*) and *J* were measured using a ferroelectric testing system (Premier-II by Radiant Technologies, Inc.). Magnetic properties were analyzed using a physical property measurement system (PPMS, DynaCool-9T, by QUANTUM DESIGN). All experimental data were recorded at ambient temperature.

## 3. Results and Discussion

**Figure 1:**
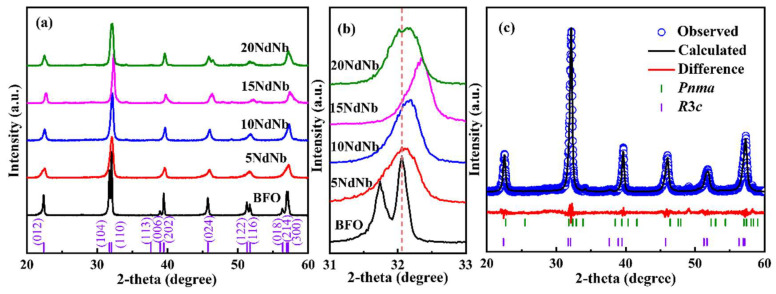
(**a**) XRD patterns of undoped and Nd–Nb co-doped BiFeO_3_ ceramics; (**b**) magnified patterns around 2θ ~32°; (**c**) Rietveld refinement XRD patterns of 10NdNb samples.

Figure 1a presents the XRD patterns of pristine and Nd–Nb co-substituted BiFeO_3_ ceramic samples. Phase-pure BF ceramics were effectively synthesized via SPS in conjunction with the powder samples obtained via the sol-gel method [24]. The diffraction peaks of (104), (110), and (113) were indexed to a characteristic perovskite structure distorted rhombohedrally with the *R*3*c* space group [24]. Impure peaks in the doped ceramics were absent, and the sharpness of the diffraction peaks indicates high crystallinity. No additional peaks were observed for ceramics up to a co-doped level of 0.10, suggesting *R*3*c* as the primary phase structure. The multiple peaks at 2θ of ~32°, 40°, 52°, and 57° merged to form a broad peak, indicating the successful doping of Nd and Nb in BF [36]. The magnified patterns at 2θ of ~32° presented in Figure 1b show the overlapping of the split peaks (104) and (110). Moreover, the peaks shifted to high diffraction angles, indicating a reduction in the unit cell size and lattice constant owing to the smaller ionic radius of Nd^3+^ than that of Bi^3+^ and the similar ionic radii of Nb^5+^ and Fe^3+^ (Nb^5+^ = 0.64 Å, Nd^3+^ = 0.983 Å, Fe^3+^ = 0.645 Å, and Bi^3+^ = 1.173 Å) [34,37]. This observation further confirms the successful integration of Nb and Nd within BF. A few peaks of 15NdNb and 20NdNb re-split to form new peaks, particularly for 20NdNb, suggesting the presence of a mixture of orthorhombic *Pnma* and rhombohedral *R*3*c* phases, conforming to the reported results [37]. Therefore, it can be inferred that Bi and Fe substituted by Nd and Nb, respectively, substantially altered the crystal structure and lattice parameters of BF.

The quantitative correlation between structural transition and doping concentration was elucidated via Rietveld refinement using the General Structure Analysis System (GSAS) program coupled with the graphical user interface (EXPGUI). Figure 1c presents the Rietveld refinement of the XRD patterns of 10NdNb. The refined lattice parameters, phase constitution, and *R*-factors of the samples are listed in Table 1. The minimal discrepancy between the observed and calculated data, along with GOF (goodness of fit, *R*_wp_/*R*_exp_) values ranging from 1.3 to 1.6, confirms the success of the refinement process. The determined *a* and *c* of the pristine BF ceramics were 5.571 and 13.872 Å, respectively, which align with the *R*3*c* space group (JCPDS No. 86-1518) [24]. The refinement considered two phases for Nd–Nb co-doped BF, such as *R*3*c* + *Pbnm*, *R*3*c* + *Pbam*, or *R*3*c* + *Pnma*. The *R*3*c* + *Pnma* model yielded the most accurate fit. The *R*3*c* fraction decreased from 97.45% to 75.12% upon Nd–Nb co-doping. Lattice parameters *a* and *c* exhibited a steady increase and decrease, respectively, which can be ascribed to the different ionic radii of Fe, Bi, Nd, and Nb and the change in bonding angle. This explains the observed shift in diffraction peaks to large angles in Figure 1b (for samples: BFO, 5NdNb, 10NdNb, and 15NdNb).

**Figure 2:**
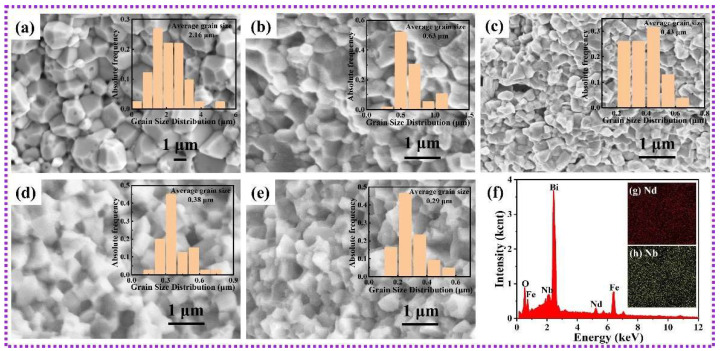
SEM micrographs of the fresh fracture surfaces for (**a**) BFO, (**b**) 5NdNb, (**c**) 10NdNb, (**d**) 15NdNb, (**e**) 20NdNb, and (**f**) EDS; (**g**) Nd element mapping; and (**h**) Nb element mapping for 15NdNb samples (inset: the average grain size).

To examine the surface morphology and grain size variations in the ceramics, SEM was utilized. Figure 2 illustrates the distinct grain structure of Nd–Nb co-doped BF samples. Analytical software (Nano Measurer 1.2, Department of Chemistry, Fudan University, Fudan, China) was employed to calculate the grain-size distributions of the as-prepared ceramics. We reported earlier that undoped BFO comprises large polyhedral grains with sizes of ~1–3 μm [24]. The obtained average grain size of BFO samples was about 2.16 μm (Figure 2a). A few pores existed at grain boundaries owing to oxygen vacancies and Bi volatilization [38]. In contrast, the Nd–Nb co-doped samples exhibited flatter boundary facets with no evident pores and a uniform distribution. The gradual reduction in grain size with increasing Nd–Nb co-doping is evident in Figure 2b–e. The grain size diminished with the increase in the co-doped content, reaching an average size <300 nm of 20NdNb, indicating that Nd–Nb co-doping effectively inhibits grain growth. It is known that the presence of vacancies in oxide materials is beneficial for ion transport during sintering and thus generates larger grains for ceramic. The marked decrease in grain size can be attributed to the low diffusivity of Nd^3+^ and the suppression of Bi volatilization and oxygen vacancy formation because of Nd–Nb co-doping [39]. Figure 2g,h illustrates the surface scan results, showing even distribution of elements within the scanned area without non-uniform aggregation or segregation. Generally, the microstructure significantly impacts the macroscopic properties, as documented in studies on ferroelectric oxides [1,2,40]. The influence of grain size on magnetic properties in BiFeO_3_ ceramics has been noted in single-phase BiFeO_3_ [41]. Consequently, with the pronounced changes in surface morphology induced by Nd–Nb co-doping, the multiferroic characteristics and electronic structure of Nd–Nb co-doped BF differ from those of the pristine BF, which is discussed in the subsequent section.

**Figure 3:**
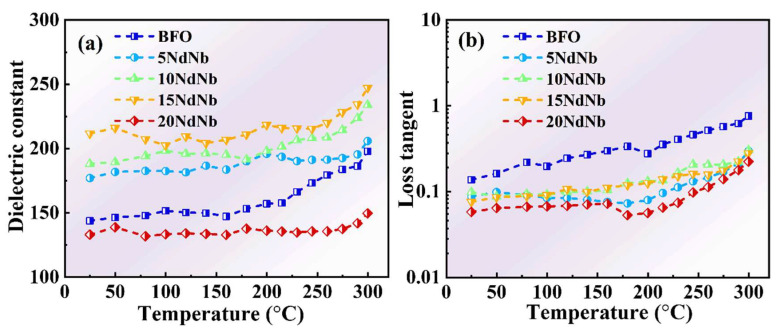
Temperature dependences of (**a**) dielectric constant and (**b**) loss tangent at 1 MHz for undoped and Nd–Nb co-doped BiFeO_3_ ceramics.

Figure 3 depicts the change in dielectric behavior (dielectric constant and dielectric loss) of pristine BF and Nd–Nb co-doped BF with temperature at 1 MHz frequency. The dielectric constant was independent of temperature below 200 °C, signifying the stability of the ferroelectric order. This stability is attributed to the high ferroelectric *T*_C_ and the unique micro- and electronic structures of BF [42]. The effect of lattice vibrations on electron movement diminished at high frequencies, leading to a less pronounced variation in dielectric properties with temperature at these frequencies. The dielectric constant increased gradually with a further increase in temperature owing to thermally activated ionic conductivity caused by an increase in defects, such as oxygen vacancies, under higher temperatures [16,19]. The loss tangent of BF increased with increasing temperature, whereas the same for Nd–Nb co-doped BF remained relatively stable below 200 °C. The dielectric constant of Nd–Nb co-doped BF increased, while the loss tangent exhibited an opposite trend to those of BF, suggesting that the dielectric characteristics of Nd–Nb co-doped BF were altered. The dielectric constants of BF, 5NdNb, 10NdNb, 15NdNb, and 20NdNb at room temperature were 143, 176, 187, 211, and 132, respectively. The SEM images of Nd–Nb co-doped BF indicate that the grain sizes of Nd–Nb co-doped samples were reduced, which increased the grain boundary. Additionally, Nd–Nb co-doping could induce phase transition and hinder Bi volatilization. Thus, resistivity was increased and the oxygen vacancy concentration was decreased [26]. Consequently, the dielectric constant was enhanced, while the loss tangent was reduced. Although the loss tangent remained slightly high, the incorporation of other ABO_3_ materials and manganese oxide (MnO_2_), or by subjecting the samples to annealing under various atmospheric conditions, could potentially have reduced the dielectric loss [43].

**Figure 4:**
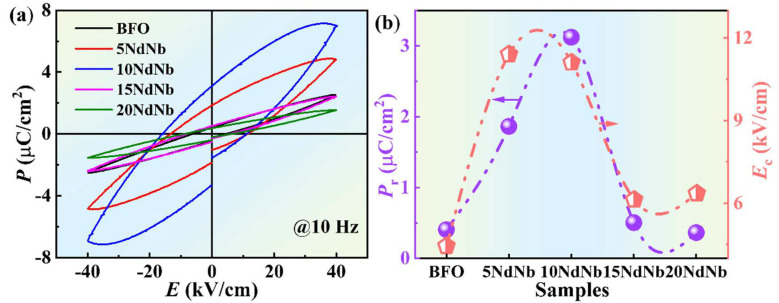
(**a**) Room−temperature ferroelectric hysteresis loops (*P*−*E*) and (**b**) ferroelectric parameters determined at 10 Hz for undoped and Nd–Nb co-doped BiFeO_3_ ceramics (*P*_r_: remnant polarization and *E*_c_: coercive field).

The ferroelectric properties of the synthesized ceramics were investigated by measuring ferroelectric hysteresis (*P*–*E*) loops at room temperature and 10 Hz using a ferroelectric analyzer [Figure 4a]. The *P*_r_ and coercivity field (*E*_c_) values of the ceramics are presented in Figure 4b and Table 2. The results were directly compared based on the magnitude, as the measurements were conducted using consistent parameters. Figure 4a shows no inflation of polarization loops, although they were not completely saturated, indicating the production of high-quality ceramics and a marked reduction in the leaky behavior of BFO. BF ceramics exhibited unsaturated and substantially inflated *P*–*E* loop area owing to a high *J* [31]. Furthermore, it is noteworthy that the ferroelectric polarization was highly dependent on the Nd–Nb concentration, with a substantial enhancement observed for the 10NdNb ceramics (see Figure 4a). Notably, *E*_c_ and *P*_r_ exhibited nonlinear behavior, as illustrated in Figure 4b. BF exhibited *P*_r_ and *E*_c_ values of ~0.41 μC cm^−2^ and ~4.45 kV cm^−1^, respectively. Nd–Nb doping substantially improved the shape of *P*–*E* loops. The *P*_r_ changed with the doping content, exhibiting an optimal value of ~3.12 μC cm^−2^ for 10NdNb, which is considerably higher than that of BF (0.41 μC cm^−2^). Thus, Nd–Nb co-doping is an appropriate strategy for enhancing the ferroelectric characteristics of BF ceramics. The substitution of Bi and Fe sites of BF by Nd^3+^ and Nb^5+^ ions, respectively, altered the grain size and structural characteristics of the crystals, affecting the *E*_c_ and *P*_r_ of the materials.

In the doped samples, some Bi sites were replaced by Nd, and the lone-pair electrons of Bi^3+^ ions (s^2^ electrons) hybridized with the empty p orbitals of Bi^3+^ or O^2−^ ions, forming a localized lobe. This phenomenon induces non-centrosymmetric distortion, thereby enhancing ferroelectricity. Nd–Nb co-doping is also responsible for changing lattice parameters. Consequently, the improved ferroelectricity was attributed to the decreased concentration of oxygen vacancy, and even the decrease in the amount of Fe^2+^, which helps to mitigate domain-pinning effects [44]. Additionally, the grain boundaries in the rhombic-phase ceramics impeded the movement of electric domains and polarization reversal, thereby enhancing ferroelectric polarization [42]. However, samples doped with highly concentrated dopants (*x* > 0.10) increased the *Pnma* phase. 10NdNb represents the optimal sample based on its position at the morphotropic-phase boundary [45]. The reduced ferroelectricity of 15NdNb might be attributed to several factors: (i) the stereochemically active 6s^2^ lone-pair electrons of Bi^3+^ and empty 6p orbits induce the ferroelectricity of BF, so when the doping content exceeds a certain value, Nd-doped Bi sites decrease the concentration of lone-pair electrons (s^2^ electrons) and deteriorate the stereochemical activity of the lone pair owing to the spherically distributed electron density of Nd^3+^ [36]; (ii) the highly centrosymmetric orthorhombic phase fails to exhibit ferroelectricity [31] and induce polarization easily; or (iii) a high Nd concentration indicates a low ability of the cations to displace within the crystal lattice, resulting in a low ion displacement and ferroelectric polarization [44]. Wu et al. [42] investigated Tb-doped BiFeO_3_ (*x* = 0.05 and 0.1) synthesized via an optimized sol-gel method and reported a maximum *P*_r_ of 6.9 μC/cm^2^. Islam et al. [38] prepared Bi_0.8_Ba_0.2_Fe_0.9_Ta_0.1_O_3_ ceramic through a solid-state reaction, achieving a *P*_r_ of approximately 0.15 μC/cm^2^. Zhang et al. [46] reported a *P*_r_ of 0.75 μC/cm^2^ for Bi_0.95_Dy_0.05_Fe_0.95_Mn_0.05_O_3_ ceramics prepared by a solid-state reaction. Hua et al. [47] reported that the *P*_r_ of Bi_0.925_Ho_0.075_Fe_0.95_Mn_0.05_O_3_ ceramic was measured to be 0.0948 μC/cm^2^. Our values are comparably favorable.

**Figure 5:**
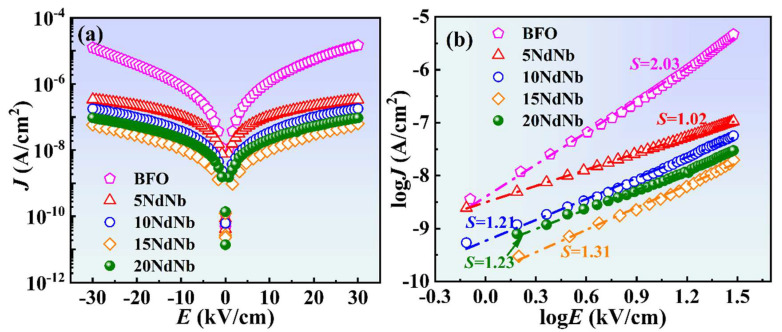
(**a**) Leakage current density as a function of electric field (*J*−*E*) and (**b**) leakage mechanism for undoped and Nd–Nb co-doped BiFeO_3_ ceramics, determined at room temperature.

Figure 5a illustrates the *J* versus *E* characteristics of the as-sintered ceramics measured at ambient temperature. The plots exhibit symmetry under negative and positive biases. The *J* of BF, 5NdNb, 10NdNb, 15NdNb, and 20NdNb ceramics at an electric field of ±30 kV cm^−1^ were ~1.26 × 10^−5^, 3.39 × 10^−7^, 1.78 × 10^−7^, 5.82 × 10^−8^, and 9.51 × 10^−8^ A cm^−2^, respectively. Thus, Nd–Nb co-doped BF ceramics were remarkably lower *J* than BF under identical electric fields. The smallest *J* of Nd–Nb co-doped BF was ~three orders of magnitude lower than that of BF, suggesting superior insulating properties. This improvement can be ascribed to the reduced Bi volatility, charge defects (oxygen or Bi vacancies), and Fe^2+^ content [2]. The bond enthalpy values of Nb−O and Nd−O were higher than those of Fe−O and Bi−O, stabilizing the perovskite structure through the substitution of Bi and Fe by Nd and Nb, respectively, which also diminished Bi evaporation [39,48]. Moreover, the substitution of Nb^5+^ ions, which had a higher valence, for Fe^3+^ ions, helped to occupy oxygen vacancies. Consequently, the co-doping of Nd and Nb effectively diminished the leakage current density in these ceramic samples.

Five primary leakage mechanisms of perovskite ferroelectric materials have been identified: Schottky emission, space–charge limited current (SCLC), Poole–Frenkel emission, ohmic conduction, and Fowler–Nordheim (FN) tunneling [35]. Specifically, ohmic conduction and SCLC are the predominant leakage mechanisms of BF-based materials. Leakage current behavior can be modeled by applying the equation $J_{\mathrm{SCLC}}\text{∝}E^{n}$. The leakage behavior follows SCLC and ohmic conduction mechanisms when exponent (n) values are 2 and 1, respectively. The leakage data were reanalyzed by plotting log *J* against log *E*, as depicted in Figure 5b. The value of n of BF was ~2, suggesting that leakage was predominantly owing to the SCLC mechanism. The slope (n) of Nd–Nb co-doped BF lay between 1 and 2, indicating combined SCLC and ohmic conduction mechanisms. Thus, the slope of BF decreased with the introduction of Nd–Nb, which implies a reduction in mobile charge carriers due to enhanced structural stability [44].

**Figure 6:**
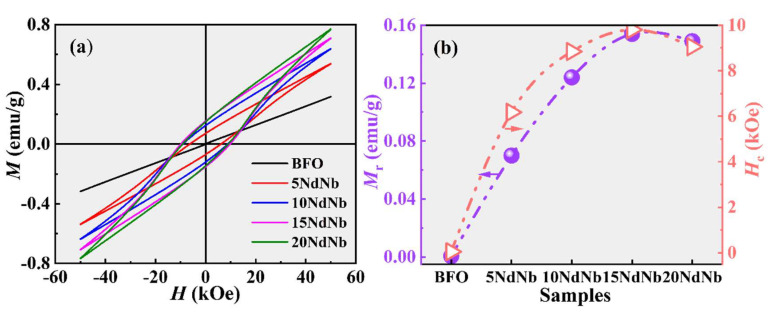
(**a**) Magnetic hysteresis loops (*M*−*H*) and (**b**) ferromagnetic parameters (remnant magnetization (*M*_r_) and coercive field (*H*_c_)) for undoped and Nd–Nb co-doped BiFeO_3_ ceramics, determined at room temperature.

Figure 6 displays the magnetic hysteresis loops of the prepared ceramic samples at ambient temperature. The *M*_r_ and coercivity (*H*_c_) data of each sample are presented in Figure 6b and Table 2. None of the loops reached saturation under the magnetic field of 50 kOe. The magnetic moment of BF was nearly negligible owing to its G-type antiferromagnetic character, similar to reported studies [49,50]. BF exhibited a nearly linear loop with a negligible *M*_r_ (~5.67 × 10^−4^ emu g^−1^), characteristic of antiferromagnetic materials, which is in agreement with other findings [49,50]. The loop centers of Nd–Nb co-doped BF expanded, indicating a shift towards weak ferromagnetism and a substantial increase in *M*_r_, suggesting a transition from an antiferromagnetic state to a ferromagnetic state [24]. The *M*_r_ values of 5NdNb, 10NdNb, 15NdNb, and 20NdNb were ~0.07, 0.12, 0.15, and 0.14 emu g^−1^, respectively, which are over two orders of magnitude higher than those of BF. This enhancement is attributed to structural distortion. Nd–Nb co-doped BF ceramics induce a structural transformation with the coexistence of rhombohedral and orthorhombic phases. The equilibrium between antiparallel spin lattices of Fe^3+^ adjacent to each other is disrupted because of the partially substituted Bi^3+^ and Fe^3+^ by Nd^3+^ and Nb^5+^, respectively, with different ionic radii and valencies. This symmetry distortion typically disrupts the spatially non-uniform spin helical structure, altering bond lengths and angles, which in turn increases the spin tilt angle and results in a net macroscopic magnetization [15]. The symmetry-driven Dzyaloshinsky–Moriya (DM) interaction mechanism suggests that the tilted antiferromagnetic spin orders demonstrate enhanced magnetic characteristics [16]. Concurrently, the unique spin–helix long-range ordered structure in BiFeO_3_ is disrupted, liberating additional magnetic moments [51], which couples with increased polarization. The high coercivity is linked to the magnetic anisotropy induced by co-doping. As documented by Monem and colleagues [52], the peak *M*_r_ for Bi_0.93_Sr_0.07_Fe_0.8_Zr_0.2_O_3_ ceramic, synthesized via a tartaric acid-assisted sol-gel method, reaches approximately 3.67 emu/g. Yadav et al. [53] and Rao et al. [39] separately synthesized Bi_0.9_Nd_0.1_Fe_1−*x*_Ti*_x_*O_3_ and Bi_0.85_Sm_0.15_Fe_0.9_Sc_0.1_O_3_ ceramics through conventional solid-state reactions, obtaining *M*_r_ values of about 3.15 and 0.2 emu/g, respectively. Evidently, the current findings are in good agreement with these studies. Without a doubt, the current results suggest that the approach of compositional design coupled with grain engineering is a promising strategy for enhancing the multiferroic properties of BiFeO_3_ at room temperature.

## 4. Conclusions

Phase-pure multiferroic Nd–Nb co-doped BF ceramics were successfully synthesized via spark plasma sintering. The phase composition of BF ceramics was altered from an exclusive *R*3*c* phase to a mixed phase comprising *R*3*c* and *Pbnm* phases via Nd–Nb co-doping. The reduced-loss tangent and *J* were correlated to the decrease in the defect concentration and refined grain size. Nd–Nb co-doping enhanced the ferroelectric, dielectric, and magnetic properties. The primary leakage mechanism of the co-doped sample was based on combined SCLC and ohmic conduction. The Bi_0.9_Nd_0.1_Fe_0.95_Nb_0.05_O_3_ ceramic sample exhibited optimal values of *P*_r_ and moderate *M*_r_ at ~3.12 μC cm^−2^ and 0.12 emu g^−1^, respectively. The improvement in ferromagnetic and ferroelectric properties of the Nd–Nb co-doped BF samples was attributed to the crystal structure, decreased leakage current, and the presence of a disrupted spiral spin configuration. These findings may offer fresh insights for advancing the multiferroic properties of BiFeO_3_ ceramics through compositional optimization and grain size manipulation, potentially paving the way for significant practical applications.

## Figures and Tables

**Table 1:** Phase constitution, lattice parameters, and *R*-factors estimated by Rietveld refinement for undoped and Nd–Nb co-doped samples.

Samples	Space Group	Phase Constitution (%)	Lattice Parameters (Å)	*R*-Factors
BFO	*R*3*c*	100	*a* = 5.571 *c* = 13.872	*R*_exp_ = 7.36 *R*_wp_ = 11.34 GOF = 1.54
5NdNb	*R*3*c*	97.45	*a* = 5.578 *c* = 13.821	*R*_exp_ = 6.57 *R*_wp_ = 8.67 GOF = 1.32
	*Pnma*	2.55	*a* = 5.623 *b* = 7.946 *c* = 5.858	
10NdNb	*R*3*c*	90.29	*a* = 5.587 *c* = 13.796	*R*_exp_ = 5.82 *R*_wp_ = 7.68 GOF = 1.32
	*Pnma*	9.71	*a* = 5.572 *b* = 7.874 *c* = 5.869	
15NdNb	*R*3*c*	85.46	*a* = 5.592 *c* = 13.774	*R*_exp_ = 6.13 *R*_wp_ = 8.65 GOF = 1.41
	*Pnma*	14.54	*a* = 5.569 *b* = 7.866 *c* = 5.883	
20NdNb	*R*3*c*	75.12	*a* = 5.597 *c* = 13.721	*R*_exp_ = 5.59 *R*_wp_ = 7.95 GOF = 1.42
	*Pnma*	24.88	*a* = 5.546 *b* = 7.810 *c* = 5.912	

**Table 2:** Ferroelectric and magnetic parameters for undoped and Nd–Nb co-doped BiFeO_3_ ceramics.

Parameter	BFO	5NdNb	10NdNb	15NdNb	20NdNb
*P*_r_ (μC/cm^2^)	0.41	1.86	3.12	0.51	0.36
*E*_c_ (kV/cm)	4.45	11.40	11.10	6.13	6.36
*M*_r_ (emu/g)	5.67 × 10^−4^	0.070	0.12	0.15	0.14
*H*_c_ (kOe)	0.050	6.17	8.84	9.80	9.05

## Data Availability

Data is contained within the article.
